# Shotgun metagenomics of fecal samples from children in Peru reveals frequent complex co-infections with multiple *Campylobacter* species

**DOI:** 10.1371/journal.pntd.0010815

**Published:** 2022-10-04

**Authors:** Craig T. Parker, Francesca Schiaffino, Steven Huynh, Maribel Paredes Olortegui, Pablo Peñataro Yori, Paul F. Garcia Bardales, Tackeshy Pinedo Vasquez, Greisi E. Curico Huansi, Katia Manzanares Villanueva, Wagner V. Shapiama Lopez, Kerry K. Cooper, Margaret N. Kosek

**Affiliations:** 1 Agricultural Research Service, U.S. Department of Agriculture, Produce Safety and Microbiology Research Unit, Albany, California, United States of America; 2 Division of Infectious Diseases, University of Virginia, Charlottesville, Virginia, United States of America; 3 Faculty of Veterinary Medicine, Universidad Peruana Cayetano Heredia, Lima, Peru; 4 Asociacion Benefica Prisma, Iquitos, Peru; 5 School of Animal and Comparative Biomedical Sciences, University of Arizona, Tucson, Arizona, United States of America; International Atomic Energy Agency, AUSTRIA

## Abstract

*Campylobacter* spp. are a major cause of bacterial diarrhea worldwide and are associated with high rates of mortality and linear growth faltering in children living in low- to middle-income countries (LMICs). *Campylobacter jejuni* and *Campylobacter coli* are most often the causative agents of enteric disease among children in LMICs. However, previous work on a collection of stool samples from children under 2 years of age, living in a low resource community in Peru with either acute diarrheal disease or asymptomatic, were found to be qPCR positive for *Campylobacter* species but qPCR negative for *C*. *jejuni* and *C*. *coli*. The goal of this study was to determine if whole-genome shotgun metagenomic sequencing (WSMS) could identify the *Campylobacter* species within these samples. The *Campylobacter* species identified in these stool samples included *C*. *jejuni*, *C*. *coli*, *C*. *upsaliensis*, *C*. *concisus*, and the potential new species of *Campylobacter*, "*Candidatus Campylobacter infans*". Moreover, WSMS results demonstrate that over 65% of the samples represented co-infections with multiple *Campylobacter* species present in a single stool sample, a novel finding in human populations.

## Author summary

Analysis of shotgun metagenomic data obtained from fecal samples of children living in a low resource tropical community of Peru revealed multiple Campylobacter species. Co-infections with more than one *Campylobacter* species within the same sample was a common finding. A potential new species of *Campylobacter* was also detected within these samples.

## Introduction

*Campylobacter* is a Gram-negative fastidious organism that requires specific atmospheric and nutritional requirements for successful culture and isolation. In high income countries, campylobacteriosis is one of the most common forms of bacterial enteritis and it is often stated that more than 95% of infections are caused by either *Campylobacter jejuni* or *Campylobacter coli* [1]. However, in low- and middle-income countries (LMICs), where campylobacteriosis is an endemic disease associated with gastroenteritis, environmental enteropathy, and growth stunting in children, disease has been shown to be associated with other *Campylobacter* species besides just *C*. *jejuni* and *C*. *coli* [2–6]. Due to challenges that limit culturing of *Campylobacter* isolates these types of infections can often be overlooked, particularly in LMICs where they are most prevalent [7].

To overcome inadequate culturing of pathogens, the Centers for Disease Control and Prevention (CDC) of the United States has highlighted the importance of culture independent diagnostic technologies (CIDTs) for the detection and quantification of foodborne bacterial illnesses [8–10]. Several CIDTs for *Campylobacter* species have been developed that are either antigen-based or nucleic acid amplification-based [11]. Indeed, large-scale epidemiologic studies have relied on CIDTs including quantitative polymerase chain reaction (qPCR), either in stand-alone assays or associated with array card technologies (BioFire, BD Diagnostics/ Enteric Bacterial Panel, Applied Biosystems), to determine infection and colonization in human samples [7,12–14]. Yet, these CIDTs target *C*. *jejuni* and/or *C*. *coli* rather than all *Campylobacter* species.

With the rapid expansion, increasing portability, and decreasing costs of next-generation sequencing (NGS), a potential sensitive alternative to PCR-based diagnostics is shotgun metagenomics. This CIDT enables the resolution of multiple *Campylobacter* species that are rarely cultured and for which specific probes have not been developed, as well as other pathogenic and non-pathogenic bacterial, viral, parasitic, and fungal populations present in a sample [15]. Additionally, it has the potential to overcome the major CIDT issue of not providing a pathogen genome for source traceback or outbreak investigations for public health agencies without reflex culturing [16,17]. Determining the genetic complexity and polyclonality of *Campylobacter* species in fecal samples from children living in highly endemic settings is a requisite for understanding transmission dynamics and attaining accurate source attribution.

Previous studies occurring in Peru have identified stool samples positive for *Campylobacter* by 16S rRNA gene-based PCR probes, and further differentiated for *C*. *jejuni* and *C*. *coli* via qPCR using the *cadF* gene. In many cases *C*. *jejuni* and *C*. *coli* were also successfully cultured from these samples. However, culturing unidentified *Campylobacter* species from stool samples positive for *Campylobacter* by 16S rRNA PCR but negative for *C*. *jejuni* and *C*. *coli* by *cadF* qPCR has yet to be consistently successful [5,13]. Thus, in this study, we utilize whole-genome shotgun metagenomic sequencing (WGMS), as a CIDT, to detect and identify the presence of other *Campylobacte*r species besides *C*. *jejuni* and *C*. *coli* in fecal samples from children in Peru.

## Methods

### Ethics statement

Samples used in this study were collected as a study approved by the Institutional Review Boards of Asociacion Benefica Prisma (Lima, Peru) and Johns Hopkins Bloomberg School of Public Health (Baltimore, MD, USA). Consent to participate in the study was obtained from the parents or legal guardians of children. Participants of both studies consented for further use of biological specimens.

### Biological samples

Archived fecal samples were derived from children enrolled in a community-based cohort study in Iquitos, Loreto, Peru. Between 2009 and 2018, 303 children were enrolled within 17 days of birth and followed for five years. Details of enrollment and surveillance procedures have been previously described [18]. Children were visited twice weekly to create a continuous daily record of early life childhood illness. Stool was collected monthly and in children experiencing diarrhea defined as >3 unformed stools in a 24-hour period. For this study we randomly chose 50 fecal samples for shotgun metagenomic sequencing out of 124 fecal samples that had previously shown to be positive for *Campylobacter* (16S rRNA gene PCR) but were negative for the *cadF* gene associated with the major thermotolerant species, *C*. *jejuni* and *C*. *coli*. These fecal samples were part of a pool of 439 symptomatic and asymptomatic fecal samples for which selection criteria has been published previously [5]. These archived samples had been stored at -80°C after initial collection.

### Nucleic acid diagnostics

Fecal DNA was extracted from 0.2 grams of feces using the QIAamp DNA Stool Mini Kit (Qiagen, Carlsbald, CA), according to the manufacturer’s instructions. A negative control consisting of RNA and DNA free water was used for each extraction set. All samples were processed using a Taqman based multiplex qPCR assay to detect *Campylobacter* spp. (16S rRNA gene), *Campylobacter jejuni* and/or *Campylobacter coli* (*cadF* gene), and *Shigella* sp. (*ipaH* gene), using the primers and probes specified in **S1 Table.** The final assay consisted of a 25 μL final reaction mixture with 12.5 μL of Environmental Master Mix (2X) (Applied Biosystems, Foster City, CA), 0.5 ul of forward and reverse primers (0.2 μM each), 0.25 μl of probes (0.1 uM each), 1 μL of DNA template and 6.75 μl of nuclease-free water (Ambion, Thermo Fisher Scientific, Waltham, MA, USA). The assays were performed on a QuantStudio 7 Flex (Applied Biosystems, Foster City, CA) using the following cycling conditions: 95°C for 10 minutes followed by 45 cycles of 95°C for 15 seconds and 60°C for 1 minutes. DNA from previously confirmed *C*. *coli* and *C*. *jejuni* were used as positive controls. Template-free controls (nuclease-free water) were included as negative controls. A cut-off cycle threshold of 38 was used to determine positivity. A sample positive for both the 16S rRNA and *cadF* genes was interpreted as positive for either *C*. *coli* or *C*. *jejuni*.

### Shotgun metagenomic DNA sequencing

Depending on the sample, 20 to 1,500 ng of DNA extracted from fecal samples was sheared in a 50 μL screwcap microtube using a Covaris M220 instrument (Covaris, Woburn, MA) at 50 peak power, 20 duty factor, 20°C, 200 cycles per burst and 25–31 seconds duration. Illumina sequencing libraries were prepared using the KAPA High-Throughput Library Preparation Kit with Standard PCR Amplification Module (Kapa Biosystems, Wilmington, MA), following the manufacturer’s instructions except for the following changes: libraries were prepared using 1/4^th^ volumes for all steps except size selected to ~500–1500 bp following double-sided size selection protocols with modified volumes of 41ul PEG/NaCL solution and 5ul AMPure XP beads. Indexing of each individual sample was done using standard desalted TruSeq HT dual index adapters from Integrated DNA Technologies (Coralville, IA) at 0.75 μM final concentration, and a total of 3–18 PCR cycles were used to minimize bias. Libraries were quantified using the KAPA Library Quantification Kit, except with 10 μl volume and 90 s annealing/extension PCR, and then pooled and normalized to 4 nM. Pooled libraries were re-quantified by ddPCR on a QX200 system (Bio-Rad, Hercules, CA), using the Illumina TruSeq ddPCR Library Quantification Kit and following the manufacturer’s protocols, except with an extended 2-min annealing/extension time. Libraries were sequenced on an Illumina MiSeq instrument (v2 500-cycle kit; Illumina, San Diego, CA) per the manufacturer’s protocols. Average sequence read lengths for each sample were >188 nucleotides. Short read data are available at NCBI SRA and are associated with BioProject PRJNA834762.

### Detection of *Campylobacter* species from shotgun metagenomics

Identification of *Campylobacter* species was performed using the Map to Reference command within Geneious Prime (v2021.2.2; Biomatters, Ltd., Auckland, New Zealand). Illumina paired-end reads >80 nt generated from an individual fecal sample were simultaneously mapped to 28 *Campylobacter* chromosomes, including: [1] *Candidatus Campylobacter infans* str. 19S00001 (CP049075.1), [2] *C*. *avium* str. LMG 24591 (CP022347.1), *C*. *canadensis* str. LMG 24001 (CP035946.1), *C*. *coli* str. 14983A (CP017025.1), *C*. *coli* plasmid pCC14983A-1 (CP017026.1), *C*. *concisus* str. ATCC 33237 (CP012541.1), *C*. *corcagiensis* str. LMG 27932 (CP053842.1), *C*. *curvus* str. ATCC 35224 (CP053826.1), *C*. *fetus* str. NCTC 10354 (CP043435.1), *C*. *gracilis* str. ATCC 33236 (CP012196.1), *C*. *helveticus* str. ATCC 51209 (CP020478.1), *C*. *hepaticus* str. HV10 (CP031611.1), *C*. *hominis* str. ATCC BAA-381 (CP000776.1), *C*. *hyointestinalis* str. CHY5 (CP053828.1), *C*. *iguaniorum* str. RM11343 (CP015577.1), *C*. *insulaenigrae* str. NCTC 12927 (CP007770.1), *C*. *jejuni* str. NCTC 11168 (AL111168.1), *C*. *lanienae* str. NCTC 13004 (CP015578.1), *C*. *lari* str. RM2100 (CP000932.1), *C*. *mucosalis* str. ATCC 43264 (CP053831.1), *C*. *pinnipediorum* str. RM17261 (CP012547.1), *C*. *rectus* str. ATCC 33238 (CP012543.1), *C*. *showae* str. ATCC 51146 (CP012544.1), *C*. *sputorum* str. LMG 7795 (CP043427.1), *C*. *subantarcticus* str. LMG 24377 (CP007773.1), *C*. *upsaliensis* str. NCTC 11541 (LR134372.1), *C*. *ureolyticus* str. RIGS 9880 (CP012195.1), *C*. *volucrus* str. LMG 24380 (CP043428.1), and *C*. *vulpis* str. 251/13 (CP041617). In total, 44 shotgun metagenomic samples with the number of reads ranging from 1,404 to 2,975,084 reads were mapped to each of the *Campylobacter* references using the low sensitivity settings (<10% mismatch between read and references), and the total number of reads mapped to each reference genome were determined. Reads mapping to genomic loci conserved between *Campylobacter* species (>90% sequence similarity between species; rRNA loci, antimicrobial genes, transposon genes, IS elements, and bacteriophage genes) were removed from the read mapping counts. These reads were determined to be non-confirmatory reads for only one species and could be mapped to multiple *Campylobacter* species or other bacterial genera. Reads were determined to be species confirmatory when BLASTn against the NCBI nucleotide (nr/nt) database resulted in the identification of a single *Campylobacter* species as the highest match, and the read was also mapped to the same *Campylobacter* species in Geneious. Additionally, the species confirmatory read should have a BLASTn >95% DNA identity across >85% of the read for a particular *Campylobacter* species. Metagenomic samples were scored as positive for a specific *Campylobacter* species when at least one species confirmatory read mapped to the genome. When <50 reads mapped to a particular genome in a sample, all reads were analyzed via BLASTn to determine if they were species confirmatory reads.

### Shotgun metagenomic data analysis

Illumina reads for an individual sample were run through the MetaWRAP pipeline [19] to characterize the entire microbial population of the sample. Initially the reads were quality trimmed and index removal using Trim Galore!(v0.6.5) and human DNA contamination was removed by mapping reads to the human GRCh37/hg19 reference genome using BMTagger [20]. Next, trimmed and cleaned reads were assembled using the metaSPAdes assembler (v3.12.0) [21], and the quality of the initial assemblies assessed using QUAST [22]. The taxonomic abundance of the initial assembled contigs and all the sequence reads were determined using Kraken2 (v2.0.8) [23,24] against the standard Kraken2 database and visualized using KronaTools [25]. Next, the metaSPAdes assembly and trimmed and cleaned reads were used to bin the contigs using both Metabat2 [26] and CONCOCT [27] programs. The initial bins were refined, screened for completeness and contamination using CheckM software (v1.0.12) [28], and consolidated into bins with at least 50% completeness and less than 10% contamination. The bins were then visualized using Blobology software [29], re-assembled using the initial refined and consolidated bins, and screened for completeness and contamination using CheckM again. Finally, the taxonomy of each contig in the different bins were assigned using Taxator-tk software [30] and annotated using Prokka [31]. Taxonomic reports generated by Kraken2 were exported as a biom file using Kraken-biom [32] and the alpha diversity, beta diversity and taxonomic profiles of the stool samples were determined using R software (v4.1.2) with the phyloseq [33], microbiome [34], and vegan [35] packages.

## Results

### Metagenomic detection of *Campylobacter* species

**Fig 1:**
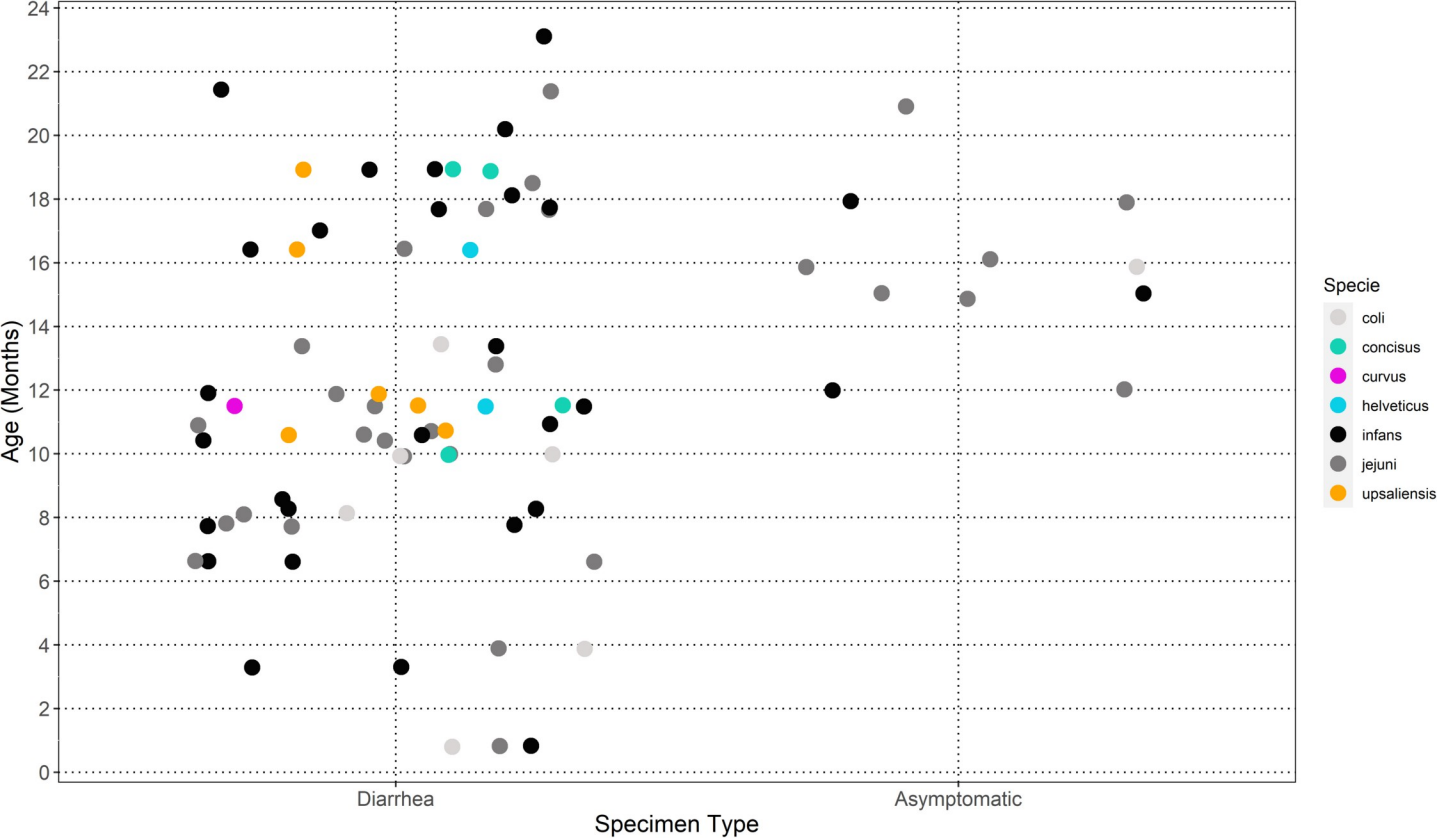
Campylobacter species in symptomatic (diarrhea) and asymptomatic (monthly) fecal samples from children under two years of age. *Campylobacter concisus*, *Campylobacter curvus*, *Campylobacter helveticus*, *and Campylobacter upsaliensis* are only found in diarrheal fecal samples from children over 10 months of age.

From the 50 archived fecal samples analyzed, we were able to successfully sequence and obtain data from 44 samples. Total DNA recovery from the 44 samples was quite variable and resulted in total reads per sample between 1,410 and 2,975,084 paired-reads, while overall 84% of samples had more than 150,000 reads (**Table 1**). The variability of DNA from the samples might be due to DNA degradation following long-term storage of the fecal samples. Nevertheless, we decided to analyze all samples with reads since previous WGMS results of infant stool samples had identified a target organism (*C*. *infans*) at >80% of all reads [36]. Mapping the sample reads against the genomes from 28 different *Campylobacter* species identified 41 out of 44 samples (93%) that were positive for *Campylobacter* reads. There were three samples in which no *Campylobacter* species was detected (97077, 118488, 130169). Despite negative qPCR results for *C*. *jejuni* and *C*. *coli* in these 44 samples, reads that matched *C*. *jejuni* (28/44 (63.6%)) and *C*. *coli* (6/44 (13.6%)) were identified in a majority of samples. Other *Campylobacter* species reads were identified in 28 samples with *C*. *infans* (26/44 (59.1%)), *C*. *upsaliensis* (4/44 (9.1%)), *C*. *concisus* (3/44 (6.8%)), *C*. *helveticus* (1/44 (2.3%)), and *C*. *curvus* (1/44 (2.3%) (**Table 1**). *C*. *concisus*, *C*. *curvus*, *C*. *helveticus* and *C*. *upsaliensis* were only identified in diarrheal fecal samples from children that were 10 months or more in age (**Fig 1**).

**Table 1:** The number of reads detected for *Campylobacter infans*, *C*. *jejuni*, *C*. *coli*, *C*. *upsaliensis*, and *C*. *helveticus*. *C*. *concisus and C*. *curvus* in fecal samples from 44 children under two years of age living in Iquitos, Peru that were positive for *Campylobacter* by a Genus specific 16S assay but negative for the *cadF* target.

Sample	Total reads	*C*. *infans*^1^	*C*. *jejuni*^1^	*C*. *coli*^1^	*C*. *upsaliensis*^1^	*C*. *helveticus*^1^	*C*. *concisus*^1^	*C*. *curvus*
13323	575,288	52 (8kb)	1,059 (281kb)	164 (27kb)			3	
13812	925,310		15		8			
15704	1,387,764	19	34		98 (10kb)	89 (6kb)	462 (83kb)	30
18102	2,975,084	72 (9kb)	66 (11kb)					
18196	8,798	5	96*					
19879	1,506,736	71,326 (1.6mb)						
22997	252,136		121	12				
25500	153,672	11,399 (488kb)	43		37			
28671	496,196		317 (39kb)	598 (86kb)				
31204	1,267,842	806 (96kb)	13		502 (63kb)			
34891	221,308	77 (9kb)	12					
37185	301,380		299 (58kb)	20				
41480	517,620		5					
52141	553,782		1,544 (258Kb)	125 (24kb)				
57052	1,253,590		139 (10kb)					
57847	1,714,028		21					
63792	392,858	4	4					
65146	14,002				8			
76987	6,862	4						
78740	18,192	4	41*					
97077	950,608							
97770	565,420	861 (154kb)					31	
97915	948,314	2						
101367	591,096		160					
108338	1,047,414	685 (68kb)	1,734 (295kb)					
118291	1,210,340	26	24					
118488	711,132							
124035	372,880		981 (179kb)					
124473	214,924	3	31					
130169	640,924							
132208	205,794	6	4					
147132	1,195,212	170 (4kb)	4166 (633kb)	404 (60kb)				
147161	1,102,270	218*						
147355	428,732	5	6					
147585	1,838,554	94 (7kb)						
148072	6,784	32						
148216	2,454,082	18	471*					
148541	2,658,540	1	446 (21kb)					
150322	246,382	105 (19kb)						
150354	1,412,860	32	104 (7kb)		6630 (350kb)	272 (16kb)		
150410	1,281,136	1	1109 (54kb)	114 (9kb)				
150583	1,410	2						
150687	1,637,790	53,637 (1.5mb)			357 (41kb)		14	
151303	11,598	12	7					

*Over representation of reads mapping at one locus, likely due to PCR amplification during library preparation.

^1^Numbers in the paratheses represent total length along a *Campylobacter* genome with >50 mapped reads. These lengths do not consider the coverage of the assemblies.

### Factors affecting species specificity of metagenomic reads

Metagenomic reads mapping to conserved genomic regions can affect specificity issues in the detection of *Campylobacter* species. The reads that mapped to the rRNA loci possessed >90% identity with multiple *Campylobacter* species due to sequence conservation of all rRNA genes. Upon BLASTn analysis, many of these mapped reads had higher scores to other genera including *Clostridium*, *Streptococcus*, and *Enterococcus*. Among the reference DNA sequences, the *C*. *coli* plasmid, pCC14983A-1 (CP017026) was included. This plasmid possesses transposon genes, a tetracycline resistance gene, and type VI secretion system genes that are common in plasmids and chromosomal loci from multiple *Campylobacter* species and other genera. There were 39 samples that had reads that mapped to the plasmid, and most reads mapped to the tetracycline resistance gene. Additionally, many of *Campylobacter* reference sequences possessed prophage, IS elements, and transposons that are shared among various *Campylobacter* species and other bacterial genera. These regions were often the only sites in certain *Campylobacter* reference sequences where reads mapped. For example, reads mapped to *tet(M)* gene and its associated mobile element of *Campylobacter lanienae* (CP015578) (**S1A Fig)**. Reads that mapped to any of these regions in a *Campylobacter* genome were determined to be non-confirmatory reads for only one species and were eliminated from further analysis **(S2 Table**).

Based on the high prevalence of *C*. *infans* in these samples and the previous findings of *C*. *infans* in stool samples from infants in various LMIC [36], the 26 *C*. *infans* positive samples were examined in detail. Fourteen of the 26 *C*. *infans* positive samples had between 1 and 32 reads mapped to the *C*. *infans* genome. BLASTn analysis of all 176 total reads from these 14 samples against the NCBI nr/nt nucleotide database resulted in identification of *C*. *infans* strain 19S00001 as the only BLASTn match, or the highest scoring match. Additionally, the reads from the 14 samples did not represent common sequence reads between these different samples. Twelve of the 26 *C*. *infans* positive samples had >50 paired-reads. Three samples 25500, 150687 (**S1B Fig, as an example)** and 19879 from diarrheal infants provided over 10,000 paired-reads mapping to the *C*. *infans* genome, resulting in approximately 1x, 5x and 9x coverages, respectively.

### Co-infection with multiple *Campylobacter* spp

The metagenomics analysis by reference assembly in Geneious detected 27 samples (65.9%) of analyzed stool with more than a single species of *Campylobacter* present in the same stool sample. Among these 27 infants that were co-infected with different *Campylobacter* species, five infants (12.2%) were co-infected with three *Campylobacter* species and one infant (3.0%) was co-infected with six *Campylobacter* species, including *C*. *jejuni*, *C*. *infans*, *C*. *curvus*, *C*. *concisus*, *C*. *helveticus* and *C*. *upsaliensis*. Three samples contained *C*. *jejuni*, *C*. *infans* and C. *upsaliensis*. Four samples were co-infected with *C*. *concisus* and *C*. *infans* (**Table 1**).

### Detection of *C*. *infans* using MetaWrap

**Fig 2:**
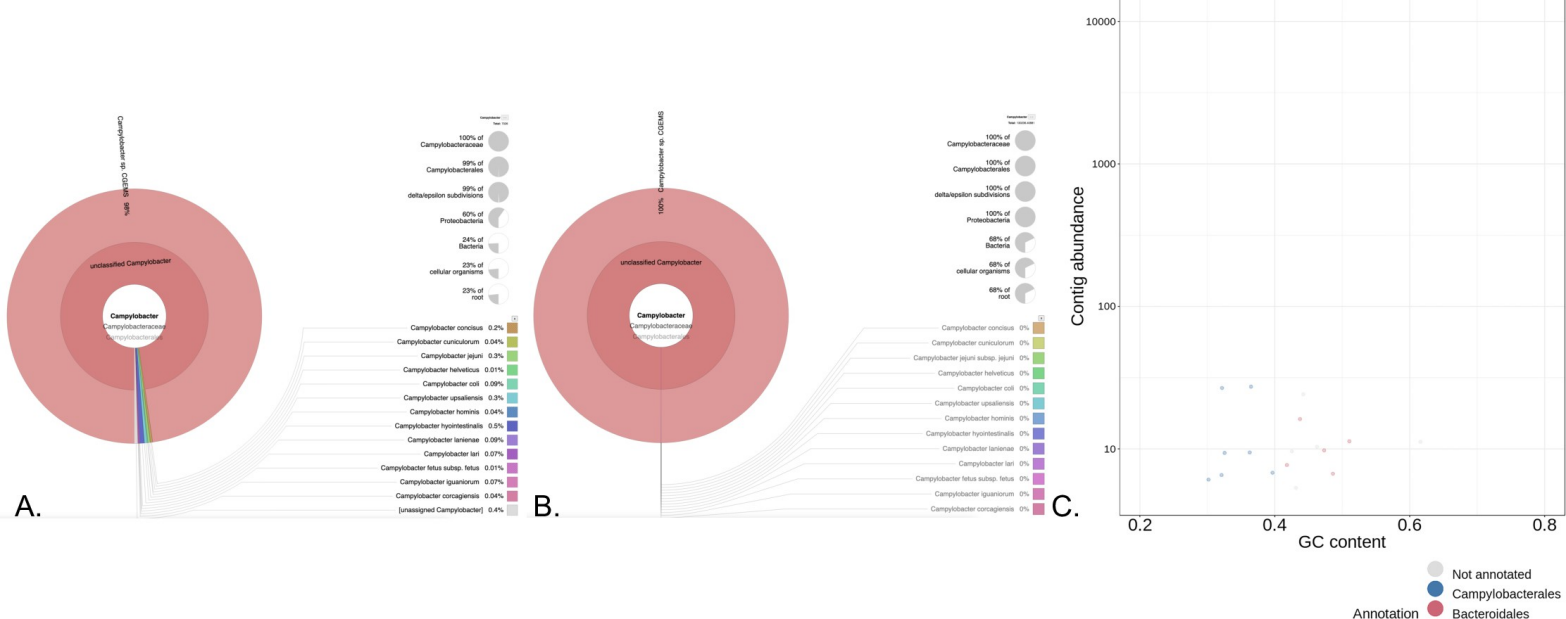
MetaWRAP analysis of sample #25500 that represents metagenomic determination of the presence of *C*. *infans* in the stool sample and provides a demonstration that a standard pipeline for analyzing whole-genome sequencing shotgun metagenomics data can identify multiple *Campylobacter* species present in the stool sample. Additionally, those sequence reads can be assembled into partial or complete genomes that can be used for source tracking or studying transmission dynamics unlike other molecular tools. **A.** Kronogram used to represent/display that 23% of total sequencing reads and 98% of the *Campylobacter* specific sequencing reads are matching *C*. *infans*, and provides a breakdown of the number of sequence reads generated for that sample the match *Campylobacter* species at the different taxonomic levels. **B.** Kronogram used to represent/display that when present in high enough concentrations as for this sample, identified *Campylobacter* species can be partially or completely genome assembled using a standard analysis pipeline and provides a breakdown of the number of assembled reads generated for that sample the match *Campylobacter* species at the different taxonomic levels. For this sample, 68% of all the assembled reads and 100% of the *Campylobacter* contigs for this sample were identified as *C*. *infans*. **C.** Shotgun metagenomic analysis pipelines can also rely on software to bin reads/contigs based on various parameters such as GC content of the data. Blobplot of all the assembled contigs that were binned from the sample based on percentage of GC content at the Order level of taxonomic identification, which demonstrates *Campylobacterales* as the dominate Order in the assembled contigs and that the binning process of the shotgun metagenomic analysis can also identify *Campylobacter* species from the stool samples. The *Campylobacter* bin contained 15 contigs that equals 20,789 bp or 1.2% of the genome.

**Fig 3:**
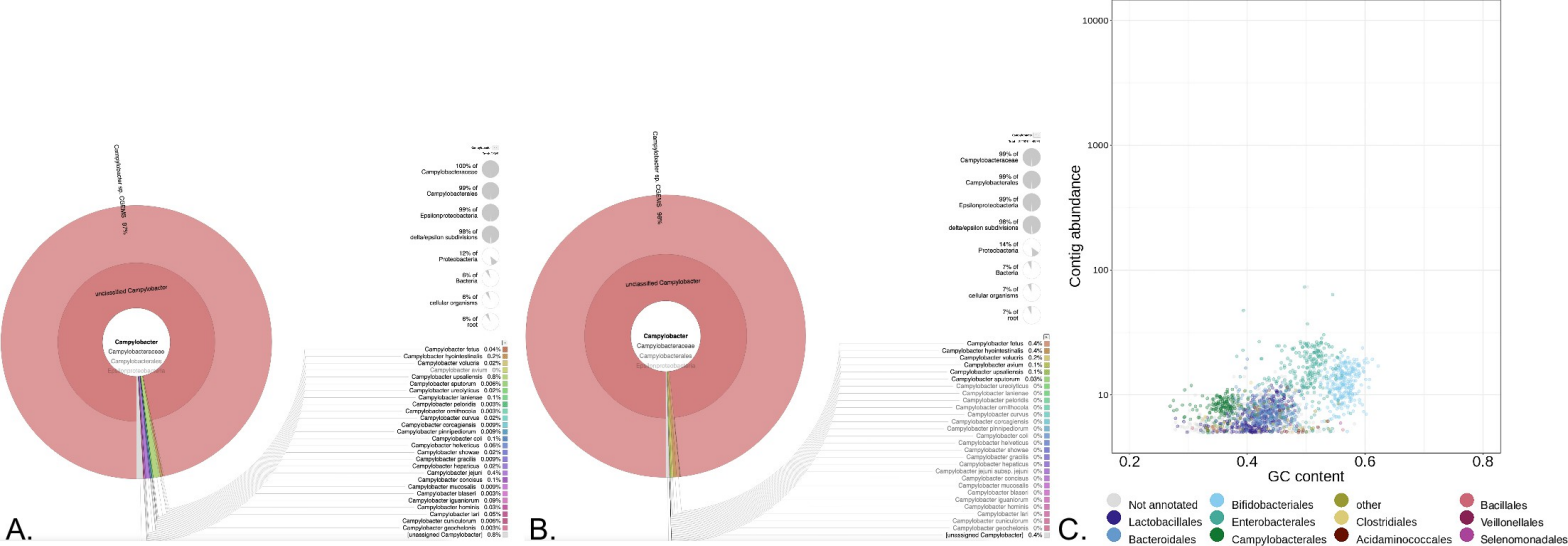
MetaWRAP analysis of sample #150687 that had lower amount of sequence reads associated with *Campylobacter*, but had a higher overall number of sequence reads generated compared to other samples in the study. Standard pipeline analysis was still able use shotgun metagenomics to determine the presence of *C*. *infans* in the stool sample and identify multiple *Campylobacter* species present in the stool sample. **A.** Kronogram used to represent/display that 6% of total sequencing reads and 97% of the *Campylobacter* specific sequencing reads are matching *C*. *infans*, and provides a breakdown of the number of sequence reads generated for that sample the match *Campylobacter* species at the different taxonomic levels. **B.** Kronogram used to represent/display that when present in high enough concentrations as for this sample, identified *Campylobacter* species can be partially or completely genome assembled using a standard analysis pipeline and provides a breakdown of the number of assembled reads generated for that sample the match *Campylobacter* species at the different taxonomic levels. For this sample, 7% of all the assembled reads and 98% of the *Campylobacter* contigs for this sample were identified as *C*. *infans*. **C.** Shotgun metagenomic analysis pipelines can also rely on software to bin reads/contigs based on various parameters such as GC content of the data. Blobplot of all the assembled contigs that were binned from the sample based on percentage of GC content at the Order level of taxonomic identification, which demonstrates *Campylobacterales* was binned during the process of the shotgun metagenomic analysis even with high levels of sequence reads not associated with *Campylobacter* and many additional bins generated from the stool sample, thus many different aspects of metagenomic analysis can identify *Campylobacter* species from stool samples. The *Campylobacter* bin contained 150 contigs that equals 1,676,492 bp or 95.6% of the *C*. *infans* genome. However, there were 171 contigs overall assembled to various *Campylobacter* species equaling 1,781,027 bp from the sample including 11 additional *C*. *infans* contigs that were not binned for a total *C*. *infans* genome of 1,740,218 bp or 99.2% of the complete genome.

Analysis of the shotgun metagenomic reads using the MetaWRAP pipeline resulted in the same identification of *Campylobacter* species reads for samples that had >500 reads mapped to a *Campylobacter* species. For samples 25500, 150687 and 19879, that possessed the most *C*. *infans* reads according to reference mapping, MetaWRAP binning modules binned (>50% completeness and <10% contamination) *C*. *infans* (*Campylobacter sp*. CGEMS) reads. According to Kraken2 module for sample 25500, 23% of all sequence reads and 68% of assembled reads were *C*. *infans* (**Fig 2**). For both samples 150687 and 19879, 6% of all reads were *C*. *infans* and 7% of assembled reads were *C*. *infans*, respectively (**Fig 3**). For other samples, MetaWRAP identified *Campylobacter* species specific reads from total reads in 36/37 (97.3%) of the samples, including *C*. *infans* as greater than 20% of the overall *Campylobacter* reads in samples 19879, 31204, 34891, 41480, 63792, 97770, 108338, 118291, 132208, 147585, and 150322, Overall, MetaWRAP analysis identified 11/37 (29.7%) of the samples had co-infections with multiple *Campylobacter* species based on two or more *Campylobacter* species associated with at least 10% of the *Campylobacter* reads each (**Table 2**).

**Table 2:** MetaWRAP analysis of stool samples from 44 fecal samples from children under two years of age living in Iquitos, Peru which were positive by PCR for the Campylobacter 16S assay but negative for the *cadF* gene.

Sample ID	Percentage of All Reads to *Campylobacter*	Percentage of All *Campylobacter* Reads				Number of *Campylobacter* contigs^1^	Number of *C*. *infans* contigs^2^	Number of *C*. *jejuni* contigs^2^
		*C*. *infans*	*C*. *jejuni*	*C*. *coli*	*C*. *upsaliensis*			
13323	0.7%	3%	32%	3%	0.3%	1 (1,074 bp)	0	1 (1,074 bp)
13812	0.004%	6%	24%	0%	41%	0	0	0
15704	0.1%	3%	2%	0%	10%	4 (9,380 bp)	0	0
18102	0.03%	42%	24%	2%	1%	0	0	0
18196	N/A	N/A	N/A	N/A	N/A	N/A	N/A	N/A
19879	6.0%	98%	0.3%	0.05%	0.3%	99 (856,787 bp)	99 (856,787 bp)	0
22997	0.2%	3%	38%	0%	3%	0	0	0
25500	23%	98%	0.3%	0.09%	0.3%	15 (20,789 bp)	15 (20,789 bp)	0
28671	0.5%	0%	19%	59%	0.7%	0	0	0
31204	0.2%	56%	2%	0.2%	36%	3 (5,689)	1 (1,209 bp)	1 (3,198 bp)
34891	0.4%	23%	2%	4%	12%	0	0	0
37185	0.2%	0%	33%	4%	0%	0	0	0
41480	0.004%	20%	20%	0%	10%	0	0	0
52141	3%	0%	39%	4%	0.4%	2 (2,047 bp)	0	0
57052	0.04%	2%	14%	3%	0%	0	0	0
57847	0.009%	4%	7%	6%	0%	0	0	0
63792	0.002%	50%	25%	0%	0%	0	0	0
65146	N/A	N/A	N/A	N/A	N/A	N/A	N/A	N/A
76987	N/A	N/A	N/A	N/A	N/A	N/A	N/A	N/A
78740	N/A	N/A	N/A	N/A	N/A	N/A	N/A	N/A
97077	0.04%	2%	19%	7%	3%	1 (1,154 bp)	0	0
97770	0.4%	64%	6%	0.4%	0.1%	4 (9,603)	2 (6,546 bp)	0
97915	0.01%	4%	4%	4%	0%	0	0	0
101367	0.2%	0.3%	74%	1%	0%	3 (4,677 bp)	0	1 (1,391 bp)
108338	0.5%	80%	3%	1%	0.6%	3 (6,989 bp)	1 (1,129 bp)	1 (3,509 bp)
118291	0.008%	34%	12%	7%	7%	0	0	0
118488	0%	0%	0%	0%	0%	0	0	0
124035	8%	0%	49%	6%	0.3%	2 (3,381 bp)	0	0
124473	0.3%	4%	42%	4%	4%	0	0	0
130169	0.01%	0%	13%	10%	0%	0	0	0
132208	0.04%	25%	0%	0%	0%	1 (1,561 bp)	0	0
147132	0.7%	0.5%	33%	4%	0.2%	13 (17.707 bp)	0	6 (9,464 bp)
147161	0.9%	5%	4%	7%	12%	143 (220,649 bp)	6 (10,651 bp)	3 (5,303 bp)
147355	0.008%	15%	23%	8%	0%	0	0	0
147585	0.02%	42%	10%	8%	3%	1 (1,134 bp)	0	0
148072	N/A	N/A	N/A	N/A	N/A	N/A	N/A	N/A
148216	0.04%	4%	3%	0%	0%	0	0	0
148541	0.03%	0.4%	42%	0.4%	0.4%	0	0	0
150322	0.2%	63%	4%	2%	1%	0	0	0
150354	0.7%	0%	0.4%	0.1%	96%	0	0	0
150410	0.2%	0%	30%	5%	0%	0	0	0
150583	N/A	N/A	N/A	N/A	N/A	N/A	N/A	N/A
150687	6.0%	97%	0.4%	0.1%	0.8%	171 (1,781,027 bp)	161 (1,740,218 bp)	0
151303	N/A	N/A	N/A	N/A	N/A	N/A	N/A	N/A

^1^Numbers in the paratheses represent total amount of *Campylobacter* sequence data from the sample that are assembled into contigs.

^2^Numbers in the paratheses represent the total amount of base pairs of the genome assembled into contigs for those *Campylobacter* species from that sample.

MetaWRAP analysis also identified other organisms in each of these samples including potential diarrheal pathogens and intestinal flora. An advantage of shotgun metagenomics is the identification of the fecal microbiome in the samples from Peruvian children with *Campylobacter* species, thus allowing additional analysis of the fecal microbiome like the impact of high *Campylobacter* abundance or number of *Campylobacter* species on the alpha and/or beta diversity of the fecal microbiome (**S2 Fig).** For infant diarrheal sample 25500, 7% of the gut microbiome was identified as *Escherichia coli* and 32% was *Bifidobacterium sp*. For both samples 150687 and 19879, *E*. *coli* reads were the larger portion of the gut microbiome at 41% and 37% and *Bifidobacterium sp*. was 16% and 0%, respectively. Besides *E*. *coli*, 67% of reads for sample 19879 were from the family *Enterobacteriaceae* including 9% reads from *Klebsiella sp*. An additional advantage of shotgun metagenomics is that each of these dominate bacteria in the fecal microbiome from the different samples can be genome assembled and binned during the pipeline analysis, as was demonstrated in the majority of the fecal samples in this study (**S3 Fig**). Having these bins then allows for further studies such as the role these commensals or other enteric pathogens have in interacting with different *Campylobacter* species in the infants intestinal tract.

## Discussion

We have previously demonstrated that stools from which *Campylobacter* is identified using qPCR, but *C*. *jejuni* and *C*. *coli* are not identified, have an elevated risk of watery diarrhea suggesting that at least some of these less well-known *Campylobacter* species result in clinical diarrhea in LMICs [5]. Herein, we demonstrated a method that identified *Campylobacter* species by mapping WSMS reads to the genomes of 27 different *Campylobacter* species using the reference assembler within Geneious Prime software, although, other assemblers could be used. Indeed, the metaWRAP pipeline retrieved similar results, identifying multiple *Campylobacter* species. The WSMS of stool samples that were previously identified as positive for *Campylobacter* by CIDT via 16S qPCR demonstrated how this second global CIDT process can broadly identify *Campylobacter* species and co-infections with multiple *Campylobacters*. We demonstrate that mapping the sample reads against the *Campylobacter* species genomes identified 41 out of 44 samples positive for *Campylobacter*. As previously observed [36], co-infections with multiple *Campylobacter* species, including *C*. *infans* and other atypical *Campylobacter* species including *C*. *upsaliensis* and *C*. *concisus* was demonstrated.

In this proof-of-principle study, we selected a group of archived samples from children who had tested positive for *Campylobacter* with a non-selective PCR probe for all *Campylobacter* species, but were negative for the *cadF* gene, one of the most common targets used to identify *C*. *jejuni* and *C*. *coli* from other *Campylobacter* species, particularly in LMICs. To our surprise, the majority of the samples contained *C*. *jejuni* and/or *C*. *coli*, despite the fact that the metagenomics and qPCR assays were run on the same extraction. This demonstrates that PCR using standard primers for the detection of the *cadF* gene is less sensitive than previously recognized.

Whole-genome shotgun metagenomic sequencing (WSMS) as a supportive, species defining CIDT strategy provides more than just confirmation of a *Campylobacter* species. First, our results demonstrate that WSMS can identify *Campylobacter* species even when the sample provides limited sequencing reads (as low as 1,400 reads), but the ability to identify the *Campylobacter* species depends on the amount of these organisms within the microbiome. When levels of *Campylobacter* were high, as seen for sample 150687 (**S1B Fig**), many distinct reads from around the genome were mapped. In fact, for samples with high read counts to a specific *Campylobacter* species, remapping the reads to diverse variable regions of the species from multiple strains, such as lipooligosaccharide and capsule biosynthesis loci [37–39], will provide additional details to the strains in the samples. At lower levels, where fewer reads were mapped, the reads discriminatory power needs to be addressed through BLASTn analysis and elimination of mapped reads that were associated with non-discriminatory regions like the rRNA loci or transposons. Species-non-confirmatory reads, such as reads mapping to rRNA loci, can map to more than one genome, and in some cases different types of bacteria. In cases where there are few reads that identify a species, species-non-confirmatory reads would require a BLASTn search, which would identify different species and reduce the level of identification accordingly. When there are species-confirmatory reads and non-confirmatory reads, the non-confirmatory reads may help validate one of species but should be used with caution to only confirm based on the species-confirmatory reads. Our study indicates that removal of bacterial conserved sequences such as rRNA loci is required to reduce false species calls. This has been described previously [40]. Finally, despite identifying *Campylobacter* species reads, samples with very few total sequence reads were not very useful for species confirmation or additional analysis since there are few confirmatory reads mapped to the pathogens being detected or to other organisms of the microbiome. Instead, samples that provide a few million reads would be more useful.

WSMS requires only a single sequencing method and given the breadth of species in the *Campylobacter* genus, a relatively efficient approach to unequivocal species identification. PCR and qPCR require distinct primer pairs for each organism to be detected and possible changes to conditions. Also, PCR/qPCR requires additional validation when altering the detectable species. With 27 possible *Campylobacter* species PCR/qPCR specific primer design quickly becomes impractical. Even designing a single 16S gene amplicon-based sequencing method requires determination and validation of new PCR conditions and primers to ensure each *Campylobacter* species may be distinguished. Using shotgun metagenomics, we utilize a method that works when there is DNA and can detect targeted organisms when there are enough reads. In this study, we were able to assemble a *C*. *infans* genome at over 95% complete for source tracking and/or transmission dynamics for one of our samples, which is useful given the nearly universal inability to culture this pathogen and lack of standardized culture protocols.

Furthermore, even validated primers like the *cadF* gene primers for *C*. *jejuni* and/or *C*. *coli* can produce false negatives, as demonstrated by the results of this study where over 60% of the samples were actually positive for *C*. *jejuni* and/or *C*. *coli* after a negative *cadF* qPCR. Third, WSMS allows multiple *Campylobacter* species, including different strains of the same species to be identified from a single sample. Many of the species within the *Campylobacter* genus are more than 10% different, so most reads only have the potential to map to the genome of one species. There are some regions that are more conserved (<10% different) between species and reads mapping to these regions are non-confirmatory. Finally, WSMS provides sequence information regarding other members of the microbiota, identify other enteric pathogens, and co-infections between *Campylobacter* and these other enteric bacterial organisms.

The most notable finding of this study was the number of samples which contained concurrent infections with multiple species of *Campylobacter*, which has recently been observed in LMIC settings [36,41]. This suggests that prolonged and persisting mixed infections are occurring in this population. The infant population under study is chronically undernourished with a high level of intestinal inflammation, which may facilitate prolonged carriage, such as the increased rate of isolation of *C*. *concisus* in patients with inflammatory bowel disease [42]. Chronic infection, in some cases over years, have been reported in immunocompromised hosts [43], and even in hosts with no evident compromised immune system as a result of microbial factors, such as a cell invasion protein [44]. Given that *Campylobacter* is closely related to *Helicobacter*, the ability of the microbe to persist in the host as a result of microbial factors is also plausible. Lastly, changes in the microbiome in the chronically infected population may be more permissive for the establishment and maintenance of *Campylobacter*. We have previously established that specific taxa in this cohort are associated with *Campylobacter* infection [44] and identified microbes that are associated with an increased risk of infection (*Ruminococcus gnavus* (ASV23), *Dialister* (ASV26), *Prevotella* (ASV204 and ASV275)) and taxa that are associated with a decreased risk of *Campylobacter* infection (*Bacteroides ovatus* (ASV40), *Ruminococcus toraues* (ASV242), *Bacteroides* (ASv27) and *Lachnospiraceae*.

*C*. *infans* was identifiable in 59.1% (26/44) fecal samples that were determined to have *Campylobacter* by a genus specific PCR assay, and that did not amplify the *cadF* gene. This potential new species of *Campylobacter* has only been isolated once from a patient in Europe, yet it was shown to be prevalent in fecal samples from children in Southeast Asia and Africa [36, 45, 46]. There is still limited information associated with the presence of this species and the occurrence of gastrointestinal diseases. Further studies are needed to inform its potential pathogenicity. Given the co-infection with *C*. *infans* and *C*. *concisus* in three infants, we hypothesize that *C*. *infans* presence may be associated with previously described decompartmentalization of the gastrointestinal tract with an increased presence of oropharyngeal flora in the stool of undernourished children [47].

Polyclonal pathogen populations facilitate the acquisition of antibiotic resistance in the infected host through the selection of resistant strains [48]. Our data suggest that co-infection of multiple *Campylobacter* species in settings of intense transmission is common and may facilitate the development of resistance and propagation of multidrug resistant *Campylobacter* in response to clinical therapeutics in LMIC at a higher rate than in the US or Europe where monoclonal *Campylobacter* infections are likely the rule. The emergence of multidrug resistant *Campylobacter* is often considered attributable principally to antibiotic usage in livestock and poultry production. However, our findings could suggest that in LMIC contexts, human antibiotic use may also play an important role but further investigation is needed to draw a definitive conclusion.

## Conclusion

WSMS was able to efficiently identify *Campylobacter* species other than *C*. *jejuni* and *C*. *coli*. Other non-*jejuni* and non-*coli Campylobacter* species are prevalent among children in Peru, of which *Candidatus* “*C*. *infans*” was the most frequently identified *Campylobacter* species, although *C*. *upsaliensis*, *C*. *concisus*, *C*. *helveticus* and *C*. *curvus* were also identified. Children in Peru infected with *Campylobacter* often have multiple species present in the intestinal tract at one time.

## Supporting information

S1 Table Primers and probes for the detection of *Campylobacter* spp., *Campylobacter jejuni/coli*, and *Shigella* spp. (DOCX)

S2 Table Nondiscriminatory Loci/Genes Identified During Analysis^a^. These loci and genes by themselves are not discriminatory for *Campylobacter* species and/or Epsilonbacteria.
(DOCX)

S1 Fig The whole-genome sequencing shotgun metagenomic reads reference from sample 150687 assembled to *Campylobacter* genomes. **A.** A portion of the *C*. *lanienae* genome (from nucleotides 275,000–294,500) possessing an example of a non-confirmatory genomic region, a mobile element. The metagenomic sequencing reads (gray rectangles below the genome map) only map across a portion of the mobile element that contains the *tet(M)* gene. The absence of reads mapping in the adjacent genomic regions is representative of the rest of the genome. B. Reads (black rectangles below the genome map) mapped to the entire *C*. *infans* genome showing read coverage of ~5X. Genomic regions containing no reads correspond to loci, such as bacteriophage, restriction modification genes, that are variably present among strains within a *Campylobacter* species.
(DOCX)

S2 Fig Fecal microbiome analysis of Peruvian children used in this study from the whole-genome sequencing shotgun metagenomic reads, all samples were rarefied to 50,000 sequence reads for all the alpha and beta diversity analysis. A. Shannon diversity of the fecal samples of children based on the number of *Campylobacter* species present in the stool sample, overall, no significant difference (p-value = (0.24). B. Bray-Curtis PCoA plot of fecal microbiome diversity based on the number of *Campylobacter* species present in the stool sample, overall, no significant difference (R^2^ = 0.1948, p-value = 0.273). C. Taxonomic barplot of the abundance of the top 12 genera present in the stool samples from the children in this study, 34/44 (77.3%) samples are represented as remaining 10 samples did not have enough reads. D. Shannon diversity of the fecal samples of children based on the overall abundance of *Campylobacter* present in the stool sample, overall, no significant difference (p-value = (0.733). E. Bray-Curtis PCoA plot of fecal microbiome diversity based on the abundance of *Campylobacter* present in the stool sample, overall, no significant difference (R^2^ = 0.0653, p-value = 0.149). High abundance ≥0.5% of all sequence reads, Low abundance ≤0.5% of all sequence reads.
(DOCX)

S3 Fig Blobplots from the binning aspect of analysis from the MetaWRAP pipeline that indicates the phylum for each bin for 32/44 (72.7%) of the stool samples, as the remaining 12 samples did not have enough sequence data to either use the MetaWRAP pipeline or generate bins. The blobplots provide overview of the number of total contigs present in each bin for a sample that were assembled for each of the samples using metaSPAdes assembler in the MetaWRAP pipeline. Thus, demonstrating even with a lower number of sequence reads for a stool sample, there is the potential for whole-genome sequencing shotgun metagenomics to result in partial or complete assemble of pathogen genomes for source tracking and transmission dynamics studies in low- and middle-income countries.
(DOCX)

S1 Data Age (months), sex and specimen type of samples included in the analysis. (CSV)
