# Supplementary material for: Shotgun metagenomics of fecal samples from children in Peru reveals frequent complex co-infections with multiple Campylobacter species
Source: PLoS Negl Trop Dis. 2022 Oct 4;16(10):e0010815. doi: 10.1371/journal.pntd.0010815 (PMC9565744; doi:10.1371/journal.pntd.0010815)
Supplement: S1 Table — (DOCX) [file pntd.0010815.s001.docx]

**S1 Table.** Primers and probes for the detection of *Campylobacter* spp., *Campylobacter jejuni/coli*, and *Shigella* spp.

| **Target** | **Nombre** | **Sequence** | Fuente |
| --- | --- | --- | --- |
| Campylobacter genus (16S rna) | 16s_Fw | 5’- CAC GTG CTA CAA TGG CAT AT -3 | PMID: **19016974** |
|  | 16s_Rv | 5’- GGC TTC ATG CTC TCG AGT T -3’ |  |
|  | 16s_Probe | 5’- /56-FAM/CAG AGA ACA /ZEN/ ATC CGA ACT GGG ACA /3IABkFQ/ -3’ |  |
| *Campylobacter coli* and *Campylobacter jejuni (cadF)* | cadF_Fw | 5’- CTG CTA AAC CAT AGA AAT AAA ATT TCT CAC -3’ | PMID: 19016974 |
|  | cadF_Rv | 5’- CTT TGA AGG TAA TTT AGA TAT GGA TAA TCG -3’ |  |
|  | cadF_Probe | 5’ -/56-VIC/CAT TTT GAC /ZEN/ GAT TTT TGG CTT GA/3IABkFQ/ -3’ |  |
| *Shigella* spp*. (ipaH)* | ipaH_Fw | 5’- CCT TTT CCG CGT TCC TTG A -3’ | PMID: 15131166 |
|  | ipaH_Rv | 5’- CGG AAT CCG GAG GTA TTG C -3’ |  |
|  | ipaH_Probe | 5’- /56-TAMN/CGC CTT TCC GAT ACC GTC TCT GCA/3IAbRQSp/ -3’ |  |
