# Supplementary material for: Shotgun metagenomics of fecal samples from children in Peru reveals frequent complex co-infections with multiple Campylobacter species
Source: PLoS Negl Trop Dis. 2022 Oct 4;16(10):e0010815. doi: 10.1371/journal.pntd.0010815 (PMC9565744; doi:10.1371/journal.pntd.0010815)
Supplement: S2 Table — These loci and genes by themselves are not discriminatory for Campylobacter species and/or Epsilonbacteria. (DOCX) [file pntd.0010815.s002.docx]

**S2 Table. Nondiscriminatory Loci/Genes Identified During Analysis^a^**

| **Loci (gene)** |
| --- |
| rRNA genes (5S, 23S, 16S) |
| Insertion sequence (IS) elements |
| *bla* gene |
| *tetM* gene |
| Glycosyltransferase genes |
| DNA methylase genes |
| Prophage genes |

These loci and genes by themselves are not discriminatory for *Campylobacter* species and/or Epsilonbacteria.
