# Supplementary material for: Shotgun metagenomics of fecal samples from children in Peru reveals frequent complex co-infections with multiple Campylobacter species
Source: PLoS Negl Trop Dis. 2022 Oct 4;16(10):e0010815. doi: 10.1371/journal.pntd.0010815 (PMC9565744; doi:10.1371/journal.pntd.0010815)
Supplement: S1 Fig — A. A portion of the C. lanienae genome (from nucleotides 275,000–294,500) possessing an example of a non-confirmatory genomic region, a mobile element. The metagenomic sequencing reads (gray rectangles below the genome map) only map across a portion of the mobile element that contains the tet(M) gene. The absence of reads mapping in the adjacent genomic regions is representative of the rest of the genome. B. Reads (black rectangles below the genome map) mapped to the entire C. infans genome showing read coverage of ~5X. Genomic regions containing no reads correspond to loci, such as bacteriophage, restriction modification genes, that are variably present among strains within a Campylobacter species. (DOCX) [file pntd.0010815.s003.docx]

**S1 Figure.** The whole-genome sequencing shotgun metagenomic reads reference from sample 150687 assembled to *Campylobacter* genomes. **A.** A portion of the *C. lanienae* genome (from nucleotides 275,000-294,500) possessing an example of a non-confirmatory genomic region, a mobile element. The metagenomic sequencing reads (gray rectangles below the genome map) only map across a portion of the mobile element that contains the *tet(M)* gene. The absence of reads mapping in the adjacent genomic regions is representative of the rest of the genome. B. Reads (black rectangles below the genome map) mapped to the entire *C. infans* genome showing read coverage of ~5X. Genomic regions containing no reads correspond to loci, such as bacteriophage, restriction modification genes, that are variably present among strains within a *Campylobacter* species.

**A.**

**
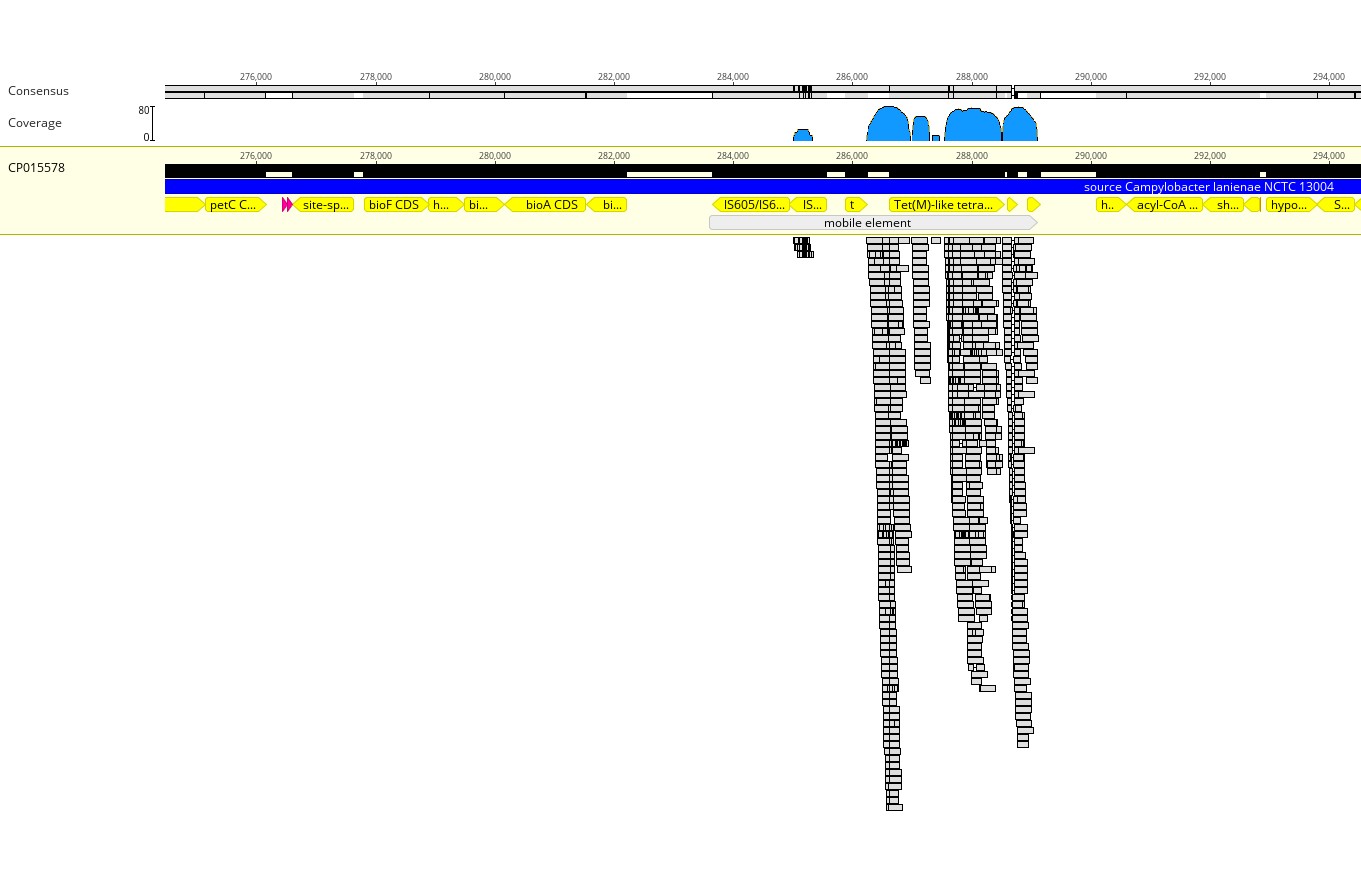
**

**B.**

**
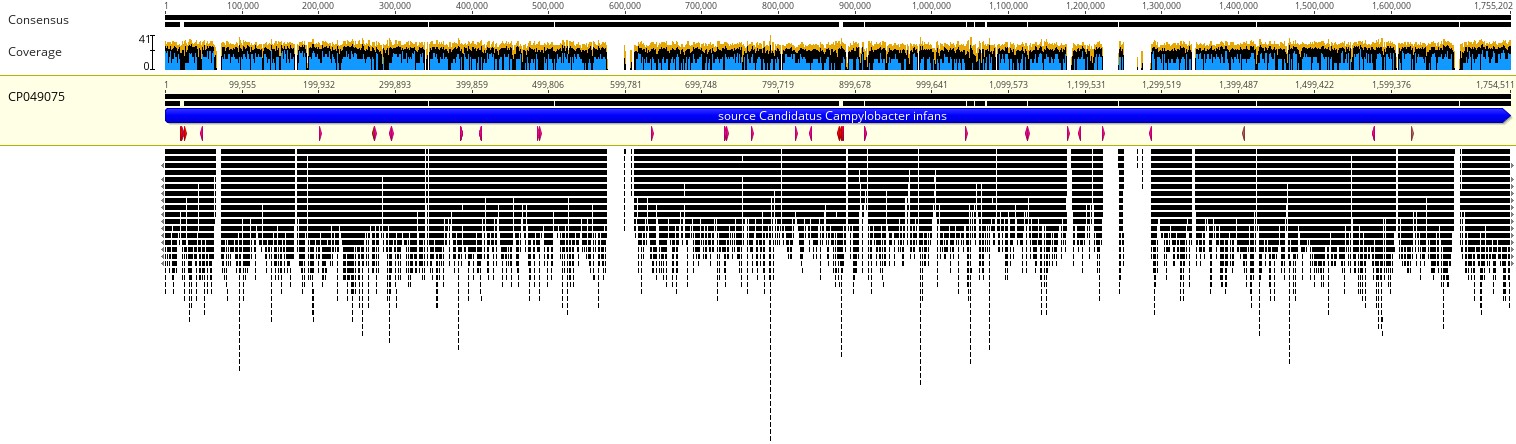
**
