# Supplementary material for: Shotgun metagenomics of fecal samples from children in Peru reveals frequent complex co-infections with multiple Campylobacter species
Source: PLoS Negl Trop Dis. 2022 Oct 4;16(10):e0010815. doi: 10.1371/journal.pntd.0010815 (PMC9565744; doi:10.1371/journal.pntd.0010815)
Supplement: S2 Fig — A. Shannon diversity of the fecal samples of children based on the number of Campylobacter species present in the stool sample, overall, no significant difference (p-value = (0.24). B. Bray-Curtis PCoA plot of fecal microbiome diversity based on the number of Campylobacter species present in the stool sample, overall, no significant difference (R2 = 0.1948, p-value = 0.273). C. Taxonomic barplot of the abundance of the top 12 genera present in the stool samples from the children in this study, 34/44 (77.3%) samples are represented as remaining 10 samples did not have enough reads. D. Shannon diversity of the fecal samples of children based on the overall abundance of Campylobacter present in the stool sample, overall, no significant difference (p-value = (0.733). E. Bray-Curtis PCoA plot of fecal microbiome diversity based on the abundance of Campylobacter present in the stool sample, overall, no significant difference (R2 = 0.0653, p-value = 0.149). High abundance ≥0.5% of all sequence reads, Low abundance ≤0.5% of all sequence reads. (DOCX) [file pntd.0010815.s004.docx]

**Supplementary Figure 2.** Fecal microbiome analysis of Peruvian children used in this study from the whole-genome sequencing shotgun metagenomic reads, all samples were rarefied to 50,000 sequence reads for all the alpha and beta diversity analysis. A. Shannon diversity of the fecal samples of children based on the number of *Campylobacter* species present in the stool sample, overall, no significant difference (p-value = (0.24). B. Bray-Curtis PCoA plot of fecal microbiome diversity based on the number of *Campylobacter* species present in the stool sample, overall, no significant difference (R^2^ = 0.1948, p-value = 0.273). C. Taxonomic barplot of the abundance of the top 12 genera present in the stool samples from the children in this study, 34/44 (77.3%) samples are represented as remaining 10 samples did not have enough reads. D. Shannon diversity of the fecal samples of children based on the overall abundance of *Campylobacter* present in the stool sample, overall, no significant difference (p-value = (0.733). E. Bray-Curtis PCoA plot of fecal microbiome diversity based on the abundance of *Campylobacter* present in the stool sample, overall, no significant difference (R^2^ = 0.0653, p-value = 0.149). High abundance >0.5% of all sequence reads, Low abundance <0.5% of all sequence reads.

**
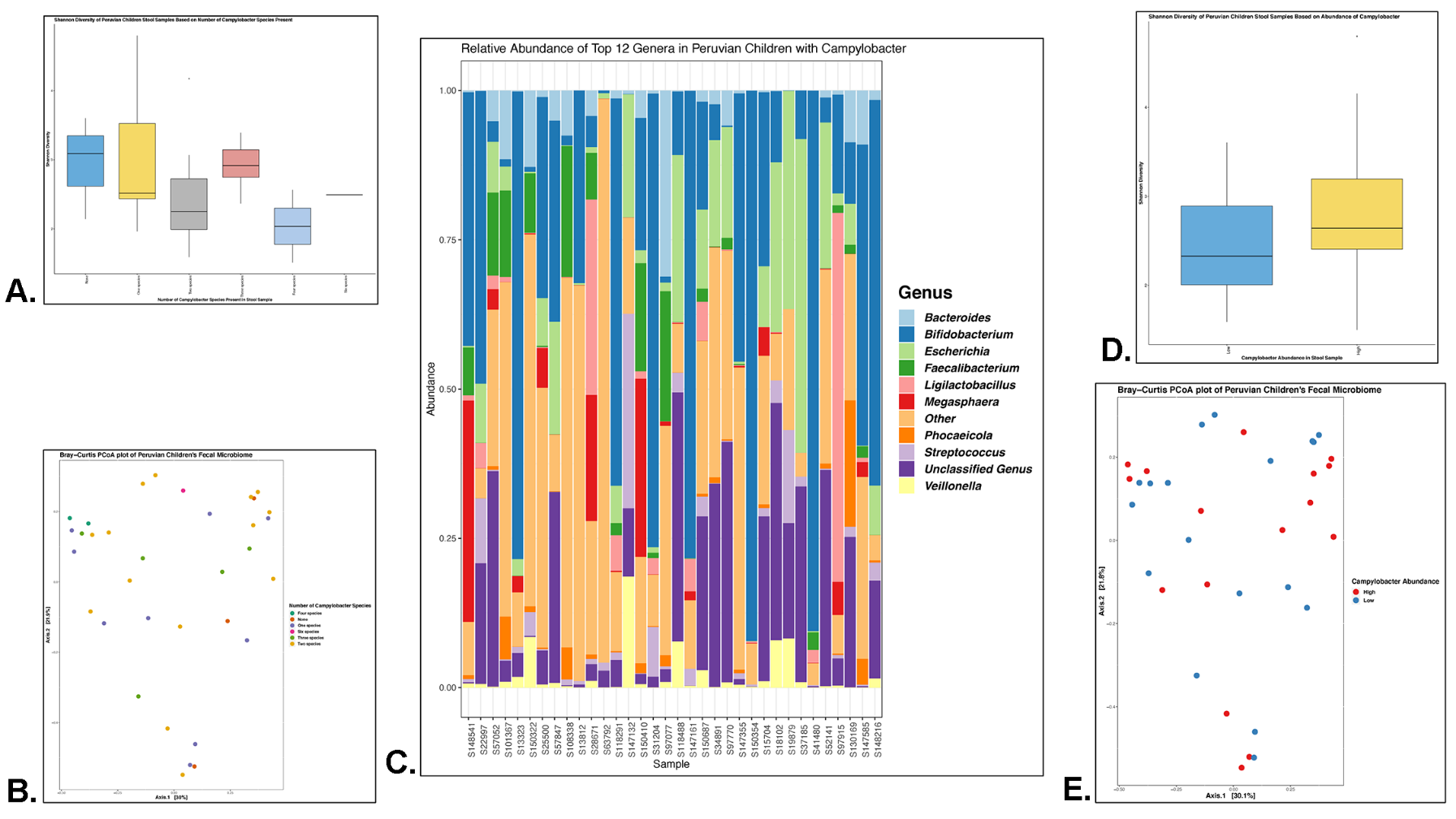
**
