# Supplementary material for: Shotgun metagenomics of fecal samples from children in Peru reveals frequent complex co-infections with multiple Campylobacter species
Source: PLoS Negl Trop Dis. 2022 Oct 4;16(10):e0010815. doi: 10.1371/journal.pntd.0010815 (PMC9565744; doi:10.1371/journal.pntd.0010815)
Supplement: S3 Fig — The blobplots provide overview of the number of total contigs present in each bin for a sample that were assembled for each of the samples using metaSPAdes assembler in the MetaWRAP pipeline. Thus, demonstrating even with a lower number of sequence reads for a stool sample, there is the potential for whole-genome sequencing shotgun metagenomics to result in partial or complete assemble of pathogen genomes for source tracking and transmission dynamics studies in low- and middle-income countries. (DOCX) [file pntd.0010815.s005.docx]

**S3 Figure.** Blobplots from the binning aspect of analysis from the MetaWRAP pipeline that indicates the phylum for each bin for 32/44 (72.7%) of the stool samples, as the remaining 12 samples did not have enough sequence data to either use the MetaWRAP pipeline or generate bins. The blobplots provide overview of the number of total contigs present in each bin for a sample that were assembled for each of the samples using metaSPAdes assembler in the MetaWRAP pipeline. Thus, demonstrating even with a lower number of sequence reads for a stool sample, there is the potential for whole-genome sequencing shotgun metagenomics to result in partial or complete assemble of pathogen genomes for source tracking and transmission dynamics studies in low- and middle-income countries.
